# Prostate-specific membrane antigen-targeted surgery in prostate cancer: Accurate identification, real-time diagnosis, and precise resection

**DOI:** 10.7150/thno.95039

**Published:** 2024-04-22

**Authors:** Jianhua Jiao, Jingjing Zhang, Weihong Wen, Weijun Qin, Xiaoyuan Chen

**Affiliations:** 1 Department of Urology, Xijing Hospital, Fourth Military Medical University, Xi'an, Shaanxi, China.; 2 Innovation Center for Tumor Immunocytology Therapy Technology, Xijing Innovation Research Institute, Fourth Military Medical University, Xi'an, Shaanxi, China.; 3 Departments of Diagnostic Radiology, Yong Loo Lin School of Medicine, National University of Singapore, Singapore, 119074, Singapore.; 4 Clinical Imaging Research Centre, Centre for Translational Medicine, Yong Loo Lin School of Medicine, National University of Singapore, Singapore, 117599, Singapore.; 5 Nanomedicine Translational Research Program, Yong Loo Lin School of Medicine, National University of Singapore, Singapore, 117597, Singapore.; 6 Institute of Medical Research, Northwestern Polytechnical University, Xi'an, Shaanxi, China.; 7 Institute of Molecular and Cell Biology, Agency for Science, Technology, and Research (A*STAR), 61 Biopolis Drive, Proteos, 138673, Singapore, Singapore.; 8 Theranostics Center of Excellenece, Yong Loo Lin School of Medicine, National University of Singapore, 11 Biopolis Way, Helios, Singapore 138667, Singapore.

**Keywords:** prostate cancer, prostate-specific membrane antigen (PSMA), molecular imaging, intraoperative, image-guided surgery

## Abstract

Radical prostatectomy (RP) combined with pelvic lymph node dissection (PLND) is the first step in multimodal treatment of prostate cancer (PCa) without distant metastases. For a long time, the surgical resection range has been highly dependent on the surgeon's visualization and experience with preoperative imaging. With the rapid development of prostate-specific membrane antigen positron emission tomography and single-photon emission computed tomography (PSMA-PET and PSMA-SPECT), PSMA-targeted surgery has been introduced for a more accurate pathological diagnosis and complete resection of positive surgical margins (PSMs) and micro-lymph node metastases (LNMs). We reviewed PSMA-targeted surgeries, including PSMA-PET-guided prostatic biopsy (PSMA-TB), PSMA-targeted radio-guided surgery (PSMA-RGS), PSMA-targeted fluorescence-guided surgery (PSMA-FGS), and multi-modality/multi-targeted PSMA-targeted surgery. We also discuss the strengths and challenges of PSMA-targeted surgery, and propose that PSMA-targeted surgery could be a great addition to existing surgery protocols, thereby improving the accuracy and convenience of surgery for primary and recurrent PCa in the near future.

## 1. Introduction

As the most frequently diagnosed cancer, prostate cancer (PCa) is the second leading cause of death in men worldwide 1. Radical prostatectomy (RP) with pelvic lymph node dissection (PLND) is the optimal choice for patients with intermediate-to high-risk PCa with or without regional lymph node metastases (LNMs) 2, 3. Accurate and complete resection of PCa lesions and positive LNMs while preserving healthy tissues as much as possible is essential but difficult, depending on the surgeon's experience in the interpretation of preoperative imaging and anatomical knowledge. Prostate-specific membrane antigen (PSMA)-targeted surgery has revolutionized traditional surgery and is a new trend in PCa surgery.

Prostate-specific membrane antigen (PSMA), also known as FOLH1 or GCP II, is a folate gamma glutamyl carboxypeptidase that is highly expressed in more than 90% of PCa lesions—including primary lesions, LNMs, and bone metastases—with 100-1000-fold higher expression level than that in normal prostate (NP) tissues 4, 5. The significantly higher PSMA expression in PCa makes it an excellent target for PSMA-targeted molecular imaging and therapies 6, 7. PSMA-targeted surgery involves using PSMA as a molecular marker to enhance the precision of PCa for an intuitive, fast and reliable way to discriminate PCa tumors from NP tissue, making the outcome of surgery less dependent on the surgeon's own experience and preoperative imaging, visual findings, or frozen sections during surgery.

PSMA-targeted surgery can be categorized as PSMA-PET-guided targeted prostatic biopsy (PSMA-TB), PSMA-targeted radio-guided surgery (PSMA-RGS), or PSMA-targeted fluorescence-guided surgery (PSMA-FGS), according to the aim and type of imaging agent.

PSMA-TB uses the precision of prebiopsy PSMA-PET to improve the accuracy of image-guided PCa biopsy 8, 9. For this purpose, PSMA-PET/MRI is the preferred imaging modality because of the better spatial resolution of MRI; PSMA-PET/CT-guided biopsy is also reliable 10, 11. For PSMA-RGS, the agent contains a PSMA-targeted ligand, linker, and gamma-emitting radionuclide, such as ^111^In-PSMA imaging and therapy (^111^In-PSMA-I&T), ^99m^Tc-PSMA imaging and surgery (^99m^Tc-PSMA-I&S) *etc.* 12, 13. For PSMA-FGS, the agent is comprised of a PSMA-targeting ligand, linker, and fluorescent probe, such as OTL78 and IS-002 *etc.* 14-16.

PSMA-RGS has been clinically applied in hospitals in Europe and Australia, and the first Phase IIa study of PSMA-FGS agents has been completed in 18 PCa patients 15. Although PSMA-FGS began its clinical trials later than PSMA-RGS, it is considered by many to be more convenient than PSMA-TB and PSMA-RGS, due to its radiation-free nature, making it popular among patients and surgeons.

PSMA-targeted surgery has the potential to replace traditional guided surgery methods for PCa. In this review, we aim to provide an overview of the status of PSMA-targeted surgery for PCa, highlighting its efficiency in detecting positive LNMs and positive surgical margins (PSMs). We also focus on patient selection before PSMA-targeted surgery and follow-up after surgery, and the several clinical questions that urgently need to be addressed.

## 2. Selecting proper candidates for PSMA-targeted surgery

As shown in Table 1, the inclusion criteria for PSMA-targeted surgeries remain unclear. In most studies on recurrent PCa, PSMA-PET is necessary, whereas the lymph node invasion nomogram is mostly used for patients with primary PCa. In fact, PSMA-PET should be conducted for each patient before prostate biopsy to perform PSMA-TB to improve the diagnostic accuracy of PSMA-PET for clinically significant prostate cancer (csPCa) 17.

**Figure 1:**
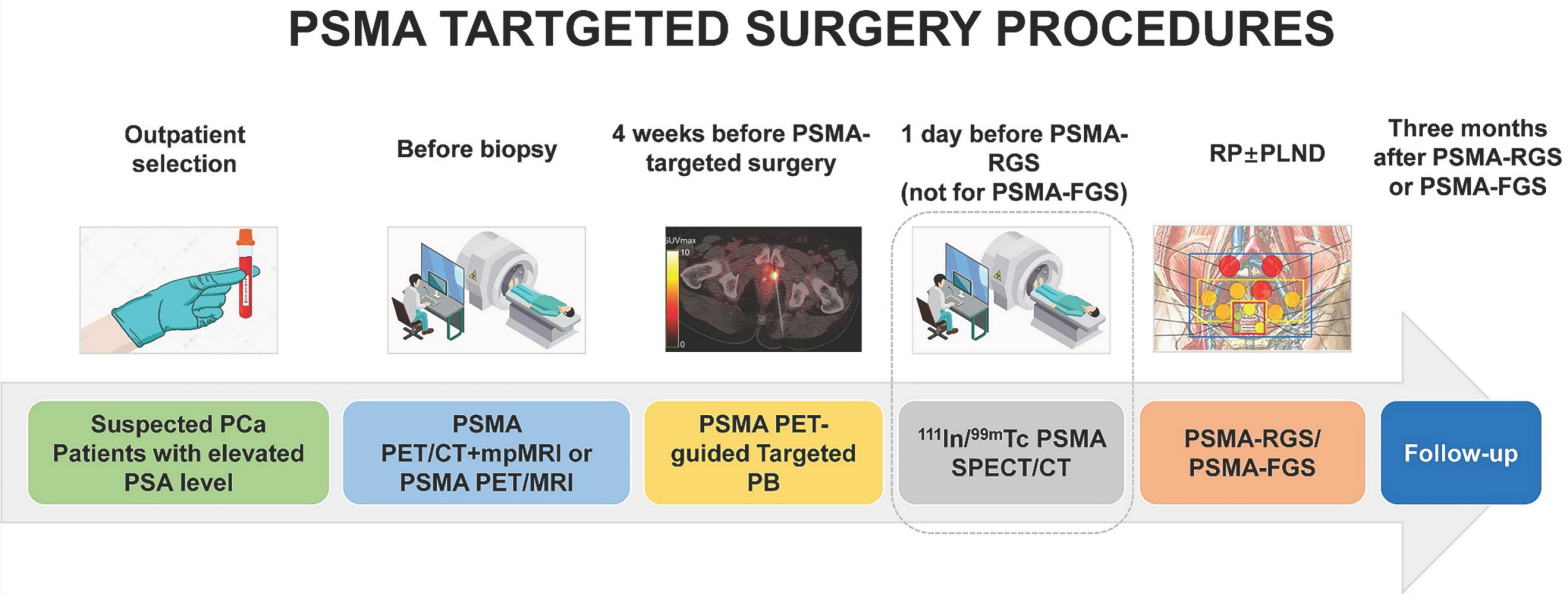
** The procedures of PSMA-targeted RGS/FGS.** The SPECT/CT on a day before PSMA-RGS can ensure that the radiotracers for PSMA-RGS have been circulated to the PCa lesions and LNMs.

Compared with the traditional viewpoint of using PSMA-PET as a staging tool after pathologically confirmed PCa, PSMA-TB may further improve the accuracy of biopsy and select suitable patients with higher uptake for PSMA-targeted surgery (Figure 1). Before PSMA-targeted surgery, patients with csPCa must be correctly diagnosed, and the presence of distant metastases (M1 stage) must be excluded. Even in patients with previously negative biopsy results, PSMA-TB can achieve a sensitivity of 100.0%, a specificity of 68.4%, a positive predictive value (PPV) of 66.7%, a negative predictive value (NPV) of 100.0%, and an accuracy of 80.6% for the detection of csPCa 18, 19. Furthermore, PSMA-PET and multiparametric magnetic resonance imaging (mpMRI) should both be used as first-line imaging modalities to diagnose and stage primary PCa before prostate biopsy (PB) 6, 7.

PSMA-PET clearly illuminates the borders of primary PCa. In comparison between PSMA-PET and robot-assisted radical prostatectomy (RARP) results, the PSMA-TB recommendation correlates better with the final histopathological results of poly(ADP-ribose) polymerase (PARP) tests. PSMA-PET can accurately detect PCa in 93.3% (28/30) and cover the highest Gleason score (GS) in 86.7% (26/30) of patients with intermediate-to-high-risk Pca 20.

Compared with mpMRI, PSMA-PET/CT has similar sensitivity (83.1% *vs.* 90.1%; p = 0.267). The missed lesions by PSMA-PET are smaller than those missed by mpMRI (0.72 cm^3^ *vs.* 1.66 cm^3^; p = 0.034) in Pca patients with similar serum prostate-specific antigen (PSA) levels, prostate volumes, and PSA density (PSAD) 10. In patients undergoing PI-RADs v2 mpMRI imaging, PSMA-PET can additionally identify 81.8% (9/11) of patients (GS ≥ 3+4) with csPCa 10. PSMA-PET/CT can also be used to predict pathological upgrading of the GS from mpMRI-targeted PB to RP, with SUV_max_ (OR: 1.223, 95% CI: 1.068-1.399, P = 0.003) as the most significant factor 21. A higher SUV_max_ (high avidity) can be used to improve the diagnosis of csPCa using PSMA-TB 22, 23.

Furthermore, compared with traditional systematic PB, PSMA-PET/CT targeted PB may improve the detection rate of clinically significant PCa with GS ≥ 7 (3+4) 11, 24. The sensitivity (100.0% *vs.* 90.9%), specificity (80.3% *vs.* 78.9%), PPV (87.9% *vs.* 78.9%), NPV (100% *vs.* 84.9%), and accuracy (92.0% *vs.* 86.2%) of PSMA-TB were higher than those of mpMRI-TB when an SUV_max_ cutoff value of 8 was used 25. In another study, PSMA-TB was performed for repeat PB in 31 patients with previously negative PB results, but persistently elevated serum PSA levels 19. PCa was found in 48.4% (15/31) of patients, 38.7% (12/31) of whom had csPCa 19. The sensitivity, specificity, PPV, NPVs, and accuracy of PSMA-PET to detect csPCa were 100.0%, 68.4%, 66.7%, 100.0%, and 80.6%, respectively 19.

In addition to PSMA-PET/CT, PSMA-PET/MRI can also be used for guided PB. In a prospective study, PSMA-PET/MRI-guided targeted PB successfully detected csPCa in 61.9% (26/42) of patients, achieving patient-based sensitivity, specificity, PPV, NPV, and accuracy of 96%, 81%, 93%, 89%, and 90%, respectively 8. In 55 patients with positive PSMA-PET results, 82.0% (146/178) and 92.7% (51/55) had PCa, and 85.5% (47/55) had csPCa 26. In a single-center study of 120 patients with elevated serum PSA levels (>4 ng/mL), transgluteal PSMA-TB detected more PCa and csPCa lesions than template PB (csPCa: 20/25 [80.0%] *vs.* 15/60 [25.0%], P < 0.01; PCa: 21/25 [84.0%] *vs.* 19/60 [31.6%], P < 0.01) 11. Similarly, in 56 patients with positive PSMA-PET-avid lesions, transgluteal PSMA-TB confirmed PCa lesions in 96.4% (54/56), of which csPCa lesions were found in 44.4% (24/54) of patients 17. PSMA-TB detected more csPCa lesions than systemic PB (81.7% *vs.* 76.7%, P < 0.001), whereas systemic PB can still have additional diagnostic benefits in 6.6% (4/60) patients 27.

For patients with regional LNMs, RP±PLND should be performed. PSMA-PET/CT can be used to exclude patients with distal LNMs (M1a stage), bone metastases (M1b stage), or visceral metastases (M1c stage) from planned curative radical prostatectomy 28. For LNMs with a diameter ≥2.3 mm, PSMA-PET can achieve a detection rate of 50.0% 29. The detection rate goes down significantly for LNMs with a diameter < 2.3 mm; small LNMs can be difficult to discover by preoperative PSMA-PET.

Therefore, PSMA-targeted surgery is needed to illuminate smaller LNMs intraoperatively and provide a chance to completely remove all primary lesions and regional LNMs. Based on the agent, PSMA-targeted surgery can be further divided into PSMA-RGS, PSMA-FGS, and multi-modality PSMA-targeted surgery.

## 3. PSMA-RGS

**Figure 2:**
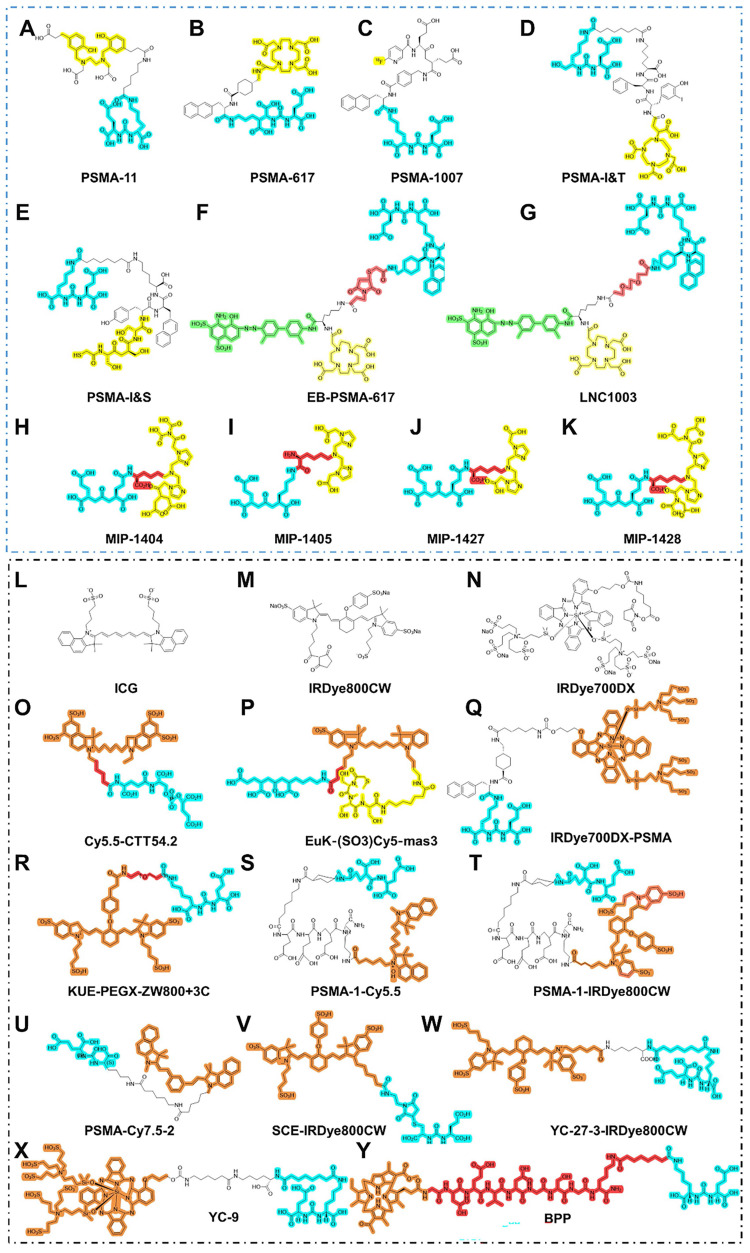
** Chemical structures of agents in PSMA-targeted RGS/FGS.** (A-K) PSMA ligands for PSMA-RGS; (L-Y) PSMA ligands for PSMA-FGS. Blue, yellow, green, red, orange areas separately highlight the PSMA binding motif, chelated area for the labeled radioisotope, albumin binder, linker, and labeled near-infrared fluorescent probe.

A series of agents have been developed for PSMA-RGS 30. For example, PSMA-I&T can be labeled with ^68^Ga for PET imaging, ^177^Lu for targeted radionuclide therapy, and ^111^In for RGS or single-photon emission computed tomography/computed tomography (SPECT/CT) 31. Mease *et al.* first established PSMA-targeted PET agent ^18^F-DCFBC and its first in-patient imaging study in five patients with metastatic PCa 32, 33. With the advent of ^68^Ga-PSMA PET for PCa, PSMA-targeted RGS was introduced to intraoperatively detect PSMA-avid LNMs with atypical localization and inconspicuous morphology. To the best of our knowledge, PSMA-targeted RGS systems, such as PSMA-I&S and PSMA-I&T, are currently the most mature methods for intraoperative navigation in clinical practice (Figure 2A-K). For theranostic purposes, PSMA ligands could be labeled with short half-life imaging isotopes, such as ^68^Ga (T_1/2_: 68 minutes), ^18^F (T_1/2_: 110 minutes), ^111^In (T_1/2_: 2.8 days), ^99m^Tc (T_1/2_: 6 hours), and for therapeutic isotopes, ^177^Lu (T_1/2_: 6.7 days], ^225^Ac (T_1/2_: 10.0 days), etc. Documented theranostic isotopes and their characteristics are shown in Table 2. We, aimed to further summarize the recent updates of PSMA-RGS according to agent type.

### 3.1 ^111^In-labeled PSMA-RGS

Although the detection rate of ^111^In SPECT/CT is lower than that of ^68^Ga-PSMA-11 PET/CT, the longer half-time of ^111^In enables ^111^In-PSMA-I&T to be appropriate for PSMA-RGS 34. Currently, most PSMA-targeted RGS systems are still highly dependent on γ-detecting probes and γ-emitting radiotracers, such as ^111^In and ^99m^Tc 35.

^111^In-PSMA is the most widely used agent for PSMA-RGS, successfully identifying 59% (29/49) PSMA-avid lesions by γ-probe. Among them, 97% of lymph nodes were confirmed as LNMs with a mean size of 7.9 mm (range, 0.8-20.0 nm) 36. The effectiveness of ^111^In-PSMA RGS is moderate due to interfering tracer accumulation in surrounding organs, leading to a more limited tumor-to-background ratio (TBR) *in vivo* compared with *ex vivo*. This issue might be mitigated by extending the time between the administration of the tracer and the PSMA-RGS procedure.

In 2015, Maurer *et al.* first traced LNMs intraoperatively with a gamma probe in five patients receiving injections of ^111^In-PSMA-I&T 24 h before surgery (Table 3) 13. Although the preliminary case report included only five patients, they successfully proved that PSMA-targeted RGS was feasible and safe 13. Furthermore, Rauscher *et al.* performed ^111^In-PSMA-I&T RGS in 31 patients with recurrent PCa, with a sensitivity of 92.3% and specificity of 93.5% 37 (Table 3). Recently, the DETECT Trial reported that ^111^In-PSMA-I&T facilitated the resection of PSMA-avid lesions, of which 97% were confirmed as LNMs in 20 newly diagnosed PCa patients 36. In addition to PSMA-I&T, another theranostic ligand, ^111^In-PSMA-617, was used for PET/CT imaging and PSMA-RGS in six primary PCa patients with potential LNMs 38 (Table 3). The sensitivity, specificity, PPV, NPV, and accuracy using cut-off SUVs normalized to lean body mass (SUL) were 92.1%, 98.9%, 94.6%, 98.4%, and 97.7%, respectively 38. In these studies, PSMA-targeted RGS could not discriminate LNMs intraoperatively within the surgery, and a γ-probe was used to confirm LNMs by counts per second (count/s, CPS) after *ex vivo* removal of the tissue samples 37, 38.

Using drop-in γ probes (DIGPs) can overcome the limited maneuverability of traditional laparoscopic gamma probes to facilitate RGS. A DIGP was designed with a 135° grip for optimal performance during robotic-assisted radical prostatectomy (RARP), while a 45° grip DIGP has been shown to be more appropriate for *ex vivo* clinical setups 39. In one study using a DIGP with nontargeted tracers (^99m^Tc-nanocolloid + ICG), 10 high-risk patients with PCa underwent sentinel pelvic lymph node dissection (PLND) with no side effects 40. In another study, with a median uptake time from injection to surgery of 5.4 h, and *ex vivo* examinations were also performed with a gamma probe and near-infrared fluorescence (NIRF) imaging 41. All LNMs were identified using DIGP and 91.0% were identified by *in vivo* NIRF imaging 41. Although sentinel PLND can detect micro-LNMs, its non-targeting nature may limit its further use, especially in patients with recurrent PCa whose lymphatic drainage has been altered by previous PLNDs. Recently, Maurer *et al.* performed a systematic comparative analysis between robot-assisted and open salvage PSMA-RGS, proving the safety and efficiency of robot-assisted PSMA-RGS with a longer operating time and equal estimated blood loss 42. Complete biochemical response (cBR, PSA < 0.2 ng/mL 2-16 weeks after PSMA-RGS) was 57% (34/61) in the open group and 61% (14/24) in the robotic group 42. The same research group further studied the reasons for negative histopathology from PSMA-RGS in 302 patients with recurrent PCa, showing that false positive lesions on PSMA-PET in most cases had negative histopathology and that only a few true-positive lymph nodes were missed by surgery 43. All true-positive lesions were adjacent to the internal iliac arteries or the prostatic fossa 43.

### 3.2 ^99m^Tc-labeled PSMA-RGS

The production and wider applications of PSMA-RGS with ^111^In-PSMA-I&T still face some limitations, such as suboptimal nuclear properties, high cost, and limited availability of ^111^InCl_3_ 12. To overcome the limitations of ^111^In-PSMA-I&T in PSMA-RGS, Robu *et al.* successfully used mercaptoacetyl triserine (MAS_3_) to replace the DOTAGA chelator in PSMA-I&T for ^99m^Tc labeling and D-Tyr-D-2-Nal to replace the 3-iodo-D-Tyr-D-Phe sequence to obtain a stronger linker 12. Compared to ^111^In, ^99m^Tc-labeled PSMA-I&T can better meet clinical needs as a more cost-effective and readily available agent. Therefore, ^99m^Tc-labeled agents were also introduced into the PSMA-RGS. The biodistributions of ^111^In labeled and ^99m^Tc labeled agents are similar, but the absorbed radiation doses can vary greatly; the total body exposure of ^111^In is 20-fold higher than that of ^99m^Tc (0.1 mGy/MBq *vs.* 0.0047 mGy/MBq) 44. Considering the total body exposure and availability of the two isotopes, technetium is the preferred choice.

Compared with ^111^In, ^99m^Tc-PSMA-RGS has been evaluated in more clinical trials, and its resolution is highly dependent on the γ-probe. The commonly used Crystal Probe CXS-SG603 has a resolution of 14 mm and is, therefore, optimally suited for identifying LNMs around 14 mm. The median diameter of correctly identified LNMs by ^99m^Tc-PSMA-RGS is 12 mm (range: 3-25 mm) 43. Compared with PSMA PET/CT, ^99m^Tc-PSMA-I&S SPECT/CT can detect only 56.8% (25/44) lesions but ^99m^Tc-PSMA-RGS can detect LNMs as small as 3 mm 45. Compared with false positive LNMs in ^99m^Tc-PSMA-RGS, true positive lesions had higher SUVmax on preoperative ^68^Ga-PSMA-11 PET [8.9 (IQR 3.8-13.8) *vs.* 2.5 (IQR 2.2-5)] 43.

In 31 patients with PCa, ^99m^Tc-PSMA-RGS yielded a sensitivity of 83.6%, specificity of 100.0%, and accuracy of 93.0%. The minimum diameter of detected LNMs was 3 mm 45 (Table 3). ^99m^Tc-PSMA-RGS has also been introduced in robot-assisted PSMA-guided salvage PLND on the robotic da Vinci^®^ platform 46. After careful measurement of occupational radiation exposure in all personnel, ^99m^Tc-PSMA-RGS was subsequently introduced in additional clinical trials 47. During the PSMA-targeted surgery with ^99m^Tc-PSMA-I&S, the measured effective dose ranged from 0 to 5 μSv, including all the personnel during labeling, quality control, syringe preparation, patient administration, patient imaging, and PSMA-targeted surgery. The mean effective doses for anesthesiology technician, scrub nurse, surgical nurse, and surgeon were 0.6 ± 0.5, 3.2 ± 1.3, 0.2 ± 0.4, and 1.0 ± 0.0 μSv, respectively. The total effective dose would be 320 μSv/year if as many as 100 procedures were performed 47; the maximum effective dose was safe for all personnel.

*In vivo* and *ex vivo* gamma probes (Crystal Probe CXS-SG603; Crystal Photonics, Berlin, Germany) were simultaneously used to remove all metastatic lesions depicted on ^68^Ga-PSMA-11 PET 45. The cutoff value to discriminate positive specimens from the background was 4 CPS 45 (Table 3). ^99m^Tc-PSMA-I&S was also validated as a safe and effective imaging technique for SPECT/CT in four healthy volunteers and 10 patients with primary PCa for initial staging 48. The injected activities of 562-828 MBq can be translated into effective doses of 3.33-4.42 mSv 48. In an *ex situ* analysis, ^99m^Tc-PSMA-I&S RGS discriminated LNMs with a sensitivity of 76.6%, specificity of 94.4%, PPV of 89.4%, and NPV of 86.9% 49 (Table 3).

In a phase 2 trial including 12 cN0cM0 primary PCa patients undergoing RARP with extended pelvic nodal dissection (ePLND), ^99m^Tc-PSMA-I&S-RGS was used to intraoperatively identify LNMs 50 (Table 3). The per-patient sensitivity, specificity, PPV, and NPV of ^99m^Tc-PSMA-I and S-RGS were 67.0%, 100.0%, 100.0%, and 90.0%, respectively 50. The per-region sensitivity, specificity, PPV, and NPV of PSMA-RGS were 63.0%, 99.0%, 83.0%, and 96.0%, respectively 50.

The main challenges in ^99m^Tc-PSMA-I&S RGS are micro-LNMs with diameters of <3 mm 50. A similar study was conducted in Australia 51 (Table 3), wherein Gondoputro *et al.* evaluated the performance of ^99m^Tc-PSMA-I&S in 12 patients with primary PCa who underwent RARP with ePLND 51. The sensitivity, specificity, PPV, and NPV were 76.0%, 69.0%, 50.0%, and 88.0%, respectively. Additionally, >90% of missed LNMs were micrometastases with diameters ≤3 mm 51. In 2022, a phase III clinical trial was performed using a DIGP with ^99m^Tc-PSMA-I&S in robot-assisted PSMA-RGS, including 20 patients with recurrent PCa 52 (Figure 6). The sensitivity and specificity of robot-assisted PSMA-RGS for the detection of LNMs were 86.0% and 100.0 %, respectively 52. In addition to PSMA-I&S, another PSMA-I&T-based analog named PSMA-I&F was also successfully conjugated to ^68^Ga/^177^Lu for targeted imaging and therapy with high affinity 53.

In summary, ^99m^Tc-PSMA-RGS has been translated into clinical use, facilitating better detection of positive LNMs than conventional ePLND in patients with PCa. The limitations of ^99m^Tc-PSMA agents include its inability to detect micro-LNMs and lesions with low to intermediate levels of PSMA. Therefore, it is important to develop new strategies to improve the imaging accuracy for better PSMA-targeted surgery. Current strategies include a combination of near-infrared dyes and dual-target methods.

### 3.3 Considerations regarding PSMA-RGS

Regarding PSMA-RGS, several key areas need further investigation to optimize patient care and outcomes. The follow-up of PCa patients receiving PSMA-RGS needs to be standardized and include an evaluation of whether positive LNMs may be missed in PSMA-RGS and their impact on prognosis. Requirements and patient selection criteria need to be established for PSMA-targeted RGS candidates, to be verified before the injection of radiotracers. Lastly, it is also critical to assess the prescribed imaging strategy, including the first preoperative imaging (^68^Ga-PSMA-PET/CT) and the subsequent second preoperative imaging (PSMA SPECT/CT) after administering the radiotracer for PSMA-RGS.

To investigate the oncological outcomes of patients with salvage PLND *via* PSMA-RGS, Knipper *et al.* showed that, in 94% (343/364) of patients receiving PSMA-RGS, 48.1% (165/343) can reach a PSA level of <0.2 ng/mL at 2-16 weeks after PSMA-RGS, with a 2-year biochemical recurrence-free survival of 32.0% and therapy-free survival of 58.0% 54 (Table 3). Adjuvant systemic therapy should be added to further improve the overall survival in patients with recurrent PCa undergoing PSMA-RGS 55. For PSMA-targeted RGS, both robot-assisted and open surgeries are appropriate for patients with recurrent PCa. Ambrosini *et al.* compared 24 patients (28%) undergoing robot-assisted RGS with 61 patients (72%) receiving open PSMA-RGS and found that they had comparable oncological outcomes with no safety concerns 42.

PSMA-PET imaging and SPECT/CT imaging should both be performed preoperatively with different purposes. PSMA-PET imaging before or after biopsy provides accurate staging, while SPECT/CT confirms the presence of PSMA-avid lesions and tracer uptake. PSMA-PET/CT can detect nearly double the number of LNMs compared with ^99m^Tc-PSMA-I&S SPECT/CT during PSMA RGS preoperative imaging 45, 56 (Figure 1). As ^99m^Tc-PSMA-I&S SPECT/CT clearly has an inferior resolution, it should only be considered for preoperative imaging when PSMA PET is unavailable 57.

As shown in Figure 1, patients with suspected PCa are recommended to undergo serum PSA testing and PSMA-PET before biopsy for PSMA-TB and accurate staging. PSMA SPECT/CT is performed after radiotracer injection to confirm specific uptake by PCa lesions. After 3 months, postoperative PSMA-PET is performed to evaluate the surgical outcome. The performance of PSMA-targeted surgery can be improved using similar approaches to those applied to PSMA-targeted theranostic agents, such as ^177^Lu-PSMA-617. Both PSMA-targeted surgery and radioligand therapy require a high tumor uptake and TBR, as well as a relatively long circulation half-life. For this purpose, albumin binders have been conjugated with PSMA-targeted radioligands to prolong blood retention time with minimal adverse effects 58. Albumin, as one of most abundant proteins in human serum, exerts an important role as a carrier protein of hormones or fatty acids 58. Its strengths, including nontoxicity, prolonged half-time, biodegradability and absence of immunogenicity enabled it as a promising candidate for theranostic agents 58. The albumin binding Evans blue (EB) moiety has proven to be a safe and effective way to improve the pharmacokinetics of PSMA-617. Compared with ^177^Lu-PSMA-617, ^177^Lu-EB-PSMA-617 accumulates significantly more in PSMA^+^ PCa tumors, reduces the needed dose of radioactivity 59, 60, is safe, and shows improved pharmacokinetics and therapeutic efficacy in 76 patients with progressive metastatic castration-resistant prostate cancer (mCRPC) 60-63. Another EB modified PSMA radioligand ^177^Lu-LNC1003 can further improve therapeutic efficacy by greatly enhancing uptake and retention, even in PCa with moderate level of PSMA expression 64, 65. Another series of dual PSMA and albumin binding ligands were also reported, such as PSMA+albumin (RPS-025, RPS-063, RPS-067, RPS-072) 66-68. For example, the dual-targeted dye RPS-027 showed higher tumor uptake and increased tumor-to-tissue distribution than MIP-1095 in PSMA-targeted α-therapy 66. Then, a series of ligands (RPS-061, RPS-063, RPS-066, RPS-067, RPS-068, and RPS-069) based RPS-027 was developed for higher tumor uptake and lower kidney uptake, but just in its preclinical models 67. Among all albumin-binding agents, LNC1003 has just finished its Phase 1 trial and showed best potential in clinical translation.

In addition to ^111^In and ^99m^Tc, ^67^Ga with three low gamma emission energy levels at 93, 185, and 300 keV has also been introduced to synthesize ^67^Ga-PSMA I&T, with the aim of overcoming the potential disadvantages of currently used radionuclides such as ^111^In, ^99m^Tc, and ^68^Ga 69 (Table 3). With a longer half-life, ^67^Ga-PSMA RGS can be performed 1 day after injection of ^67^Ga-PSMA I&T, with the option of a further delay of 1 or 2 more days 69.

To improve the diagnostic efficiency of PSMA-targeted agents, a series of glutamate-urea-glutamate or glutamate-urea-lysine pharmacophores incorporating second-generation single amino acid chelators were developed for ^99m^Tc-labeled PSMA SPECT imaging, such as MIP-1404, MIP-1405, MIP 1428, and MIP-1427 70, 71. Among them, MIP-1404 achieved the best binding affinity by PCa tumors (1.07 ± 0.89 nM) with rapid clearance from the kidneys and non-tumor tissues 70. Compared with MIP-1405, MIP-1404 showed high-contrast imaging and lower urinary activity (7% *vs.* 26%) in six healthy men and six patients with metastatic PCa 72. ^99m^Tc-MIP-1404 has shown good accuracy in the detection of PCa lesions and LNMs; however, it has not been used in PSMA-RGS. ^99m^Tc-MIP-1404 was first tested in four clinical trials involving 39 patients 73. In a multicenter phase 2 clinical trial including 105 intermediate- to high-risk PCa patients, ^99m^Tc-MIP-1404 detected 93.3% (98/105) of primary PCa patients, with a sensitivity of 50.0% and a specificity of 87.0% for the detection of LNMs 74. Similar results were reported in another study in 2018, wherein Schmidkonz *et al.* performed ^99m^Tc-MIP-1404 SPECT/CT in 93 patients with pathologically confirmed primary PCa, and detected 96.7% (90/93) of LNMs 75. TU_prostate_ was significantly higher in patients with a GS > 7 or PSA > 10 ng/ml 75. In 60 biochemically recurrent PCa patients, ^99m^Tc-MIP-1404 SPECT/CT could detect 91.4% of PCa patients with PSA ≥ 2 ng/ml and 40.0% of PCa patients with PSA < 2 ng/ml 73. In a larger study including 225 PCa patients with biochemical recurrence, detection rates were 90.0% in patients with PSA ≥ 2 ng/mL and 54.0% in patients with PSA < 2 ng/mL, leading to treatment management in 74.0% of the patients 76. In biochemically recurrent PCa patients with PSA 0.2-0.5 ng/mL, the detection rate of ^99m^Tc-MIP-1404 SPECT/CT was 44.0% 77.

Although ^99m^Tc-MIP-1404 has not been used in PSMA RGS, it has potential to become the next ^99m^Tc-labeled agent for PSMA RGS. Compared with other SPECT/CT tracers, ^99m^Tc-MIP-1404 has better imaging performance, achieving a similar lesion-to-liver ratio to ^68^Ga-PSMA-11 PET in patients with mCRPC (median, 18.2 *vs.* 17.3; P = 0.93) 78. With favorable radiation dosimetry, compared to ^68^Ga-PSMA-11 (0.0088 mSv/MBq *vs.* 0.022 mSv/MBq), ^99m^Tc-MIP-1404 has shown imrpoved efficacy in patients with intermediate-to-high-risk PCa 74, 78. ^99m^Tc-MIP-1404 could be used as a secondary alternative when PSMA-PET/CT is unavailable.

PSMA PET/CT has also been used for intraoperative imaging 79. Intraoperative PET/CT imaging (AURA10; XEOS Medical, Gent, Belgium) was first used to detect specimens from RP and lymphadenectomy *ex vivo* in a prospective two-center feasibility study 80, 81. This technique can provide a quicker method to detect PSMs in resected specimens than frozen section analysis; however, this type of imaging also has obvious shortcomings. The *ex vivo* specimen PET/CT imaging cannot determine whether all positive lesions (prostatic lesions and LNMs) have been totally removed by surgery because no postoperative PSMA PET/CT is performed, whether the estimated dose per procedure is safe for personnel, and whether the *ex vivo* imaging can correctly match the final pathological results of the whole prostate. Therefore, we believe that *ex vivo* PET/CT imaging of specimens should not be the preferred method for PSMA-RGS.

## 4. PSMA-FGS

### 4.1 PSMA-FGS in clinical stage

Compared to PSMA-RGS, PSMA-FGS is a more promising and attractive strategy for intraoperative navigation because it is more intuitive. It is dynamically fitted for the da Vinci surgical system, equipped with a fluorescent imaging system. Currently, OTL78 and IS-002 are two agents for PSMA-FGS that have entered clinical trials 14-16 (Table 4). The median size of correctly detected LNMs by PSMA-FGS-using OLT 78 was 1.9 mm (IQR 1.8-2.8), much smaller than that of PSMA-RGS. The exact resolution of IS-002 is yet to be determined. The sensitivity of *ex vivo* imaging with OLT78 was higher than its *in vivo* imaging, because the limited range of motion of a laparoscope and limited range of fluorescence may cause difficulties in the in vivo visualization of the apex of the prostate, which is located in the deep region of pelvis 15. Compared with the laparoscope, robotic surgery systems may greatly facilitate deep pelvic visualization and can be better integrated into a robot-assisted fluorescence-guided surgery workflow.

In a single-arm, phase 2a, feasibility trial, Stibbe *et al.* showed that OTL78 was well tolerated and had the potential to achieve complete oncological resection of PSMs and positive LNMs, with the optimal dose of 30 µg/kg at 24 h preoperatively (Figures 4-5) 15. Similarly, Nguyen *et al.* conducted a phase 1, single-center, dose-escalation study to evaluate another near-infrared agent, IS-002, in 24 men with high-risk PCa by RP+ePLND 16. The PSMA-FGS with IS-002 achieved a PPV of 97% and NPV of 45% for LNMs, and PPV of 100% and NPV of 80% for locoregional/residual disease detection with an injection dose of 25 µg/kg (Figures 4-5) 16. These studies using OTL78 or IS-002 demonstrate the excellent performance of PSMA-FGS in the detection of PCa lesions and LNMs, as clinicians prefer real-time imaging and radiation-free agents.

### 4.2 PSMA FGS in the preclinical stage

In addition to OTL78 and IS-002, other agents for PSMA FGS are still in their respective preclinical stages (Figure 2L-W). Based on the binding motif, PSMA-targeted agents in FGS can be divided into antibody-conjugated NIRF dyes, small-molecule-conjugated NIRF dyes, and activable NIRF dyes.

#### 4.2.1 Antibody-conjugated NIRF agents

In 2011, a humanized anti-PSMA antibody, huJ591, was conjugated with indocyanine green (ICG); however, huJ591-ICG was quenched until uptake by PSMA^+^ PCa tumors 82 (Table 5). The quenching ability of huJ591-ICG was mainly due to the unique property of ICG, which loses its fluorescence when bound to the antibody, but regains fluorescence when it is separated from the antibody after internalization and degradation of the ICG conjugate in the lysosome 82. Despite the advantages of ICG, such as documented safety and deep tissue penetration, the fluorescence quenching could hinder the visibility and identification of target areas. To maintain fluorescence and bypass the quenching ability of ICG conjugates, a series of IRDyes were developed and conjugated with huJ591. The IRDye800CW is a hydrophilic dye that can be easily synthesized and maintained *in vitro* and *in vivo*. IRDye800CW was introduced to replace ICG to bind huJ591 as ProsaFluor (huJ591-ICG-IRDye800CW) 83 (Table 5). Unlike IRDye800CW, IRDye700DX is another dye that could provide simultaneous NIRF imaging and NIR-infrared photoimmunotherapy, where IRDye700DX, upon activation by NIR light of a wavelength that correlates to the absorption spectrum of the agent, produces reactive oxygen species (ROS) and thus induces localized ROS-mediated cell death in targeted cells. IRDye700DX is a hydrophilic dye that differentiates it from previous hydrophobic dyes, with associated advantages including easier distribution throughout the body without the need for complex solubilizing agents, no aggregation in aqueous solutions leading to less non-specific binding to various tissues, facilitated conjugation with targeting molecules, and therefore overall better biocompatibility. Both IRDye800CW and IRDye700DX have been approved by the US Food and Drug Administration (FDA) to start clinical trials, indicating their safety for use in FGS.

Nagaya *et al.* incubated a fully human anti-PSMA antibody with IRDye700DX to develop anti-PSMA-IR700, showing the potential of PSMA-targeted FGS and near-infrared photoimmunotherapy (NIR-PIT) 84 (Table 5). Chen *et al.* synthesized a PSMA-targeted theranostic photosensitizer, YC-9, by conjugating IRDye700DX to a PSMA-targeted Lys-Glu urea-based molecule for NIR-PIT 85. Furthermore, they developed a long-circulating PSMA-targeted bacteriochlorophyll-based photosensitizer (BPP) for NIRF imaging within surgery and NIR-PIT 86. To further improve its clinical use, another fully human anti-PSMA antibody, named PSMAb, based on a PSMA-specific single-chain variable fragment (scFv) named gy1 87, 88 (Table 5), was used. Both PSMAb and gy1 have been labeled with IRDye800CW to illuminate PSMA^+^ PCa tumors 87, 88. ScFvD2B, another high-affinity anti-PSMA scFv, was conjugated to X770 (an NRIF probe) 89 (Table 5) with IRDye700DX, DTPA, and ^111^In to develop the multi-modality agent, DTPA-D2B-IRDye700DX 90 (Table 5).

Antibodies or antibody fragment conjugates can be used for the optical imaging of PSMA-positive tumors. The advantages of antibody conjugates include a long circulation half-life, providing a long time window for FGS, and reliable safety because most of the antibodies have been approved by the FDA for treatment in clinical trials. However, due to the nonspecific uptake of antibodies by the liver and spleen, only a small portion of the conjugates are available to bind the PSMA-positive tumor. Therefore, for PSMA-FGS, a focus has instead been placed on small-molecule conjugated NIRF agents.

#### 4.2.2 Small molecule-conjugated NIRF agents

Small-molecule inhibitors include CTT-54.2, YC273, PSMA-1, SCE, KUE, and OTL78 14, 91-95 (Table 5). Cy5.5-CTT-54.2 displayed high targeting potency for PSMA, but *in vivo* studies are lacking 91. Similarly, YC-273-IRDye800CW, PSMA-1-IRDye800CW, PSMA-1-Cy5.5, and SCE-IRDye800CW have been established and preliminarily validated in PSMA^+^ PCa tumor-bearing mice 92-94. In addition to polymethine fluorophores, Bao *et al.* reported a zwitterionic (ZW) fluorophore conjugated directly with a urea-containing PSMA ligand, lysine-urea-glutamate (KUE), ZW800-1, for the NIRF imaging of PSMA^+^ PCa tumors 95. Kularatne *et al.* designed and synthesized OTL78, which has a circulation half-life of 17 min and excellent TBR (5:1) for PSMA-targeted NIRF surgery 14. Chen *et al.* reported that PSMA-targeted NIRF dyes for PSMA FGS and DyLight800-10 achieved the highest uptake in PCa tumors 96 (Table 5). Furthermore, Yang *et al.* conjugated an iridium(III) complex with the inhibitor Lys-urea-Glu to create a PSMA-targeted iridium(III) probe 97.

Preoperative ^68^Ga-PSMA PET/CT and ICG-guided PLND were combined in early studies, achieving a sensitivity of 92.8% and specificity of 39.1% 98. However, due to ICG by itself not being tumors-specific, the specificity of PLND was low. To develop PSMA-targeted NIRF dyes for guided surgery, several studies have attempted to develop PSMA-targeted ligands using PSMA-PET/CT. Eder *et al.* also developed a PSMA-targeted NIRF dye, Glu-urea-Lys-2-Nal-Chx-Lys (IRDye800CW)-DOTA (PSMA-927), based on the theranostic agent PSMA-617 99 (Table 5). Moreover, PSMA-Cy7.5-2 outperformed PSMA-IRDye800CW *in vitro* and *in vivo* 100.

To evaluate the performance of the PSMA-targeted agents, PSMA-negative PC3 cells were transfected with a PSMA-expressing lentivirus to obtain PC3-PSMA^+^ cells, which were used to evaluate the efficacy of PSMA-targeted NIRF dyes. Similarly, IRDye700DX was also conjugated to PSMA-617 as IRDye700DX-PSMA for preliminary validation in PC3-PSMA^+^ tumor mice 101. Derks *et al.* modified the PSMA peptide linker from glutamic acid to a lysine residue and synthesized three dual-modality DOTA(GA)-IRDye700DX-PSMA ligands (PSMA0N01, PSMA-N02, and PSMA-N03) based on the existing PSMA backbone structures (PSMA-1007/PSMA-617) 102 (Table 5). Dell'Oglio *et al.* developed and validated the performance of intraoperative PSMA-mediated FGS using EuK-(SO_3_)Cy5‐mas_3_ in a porcine model, demonstrating its clinical translational potential 103 (Table 5). However, while the porcine model enables the study of robot-assisted radical RP within a large animal model, unlike humans or mice, it does not have PSMA-positive tumors; therefore, targeted agents still need further evaluation in mouse models and clinical trials.

#### 4.2.3 Activatable NIRF dyes

**Figure 3:**
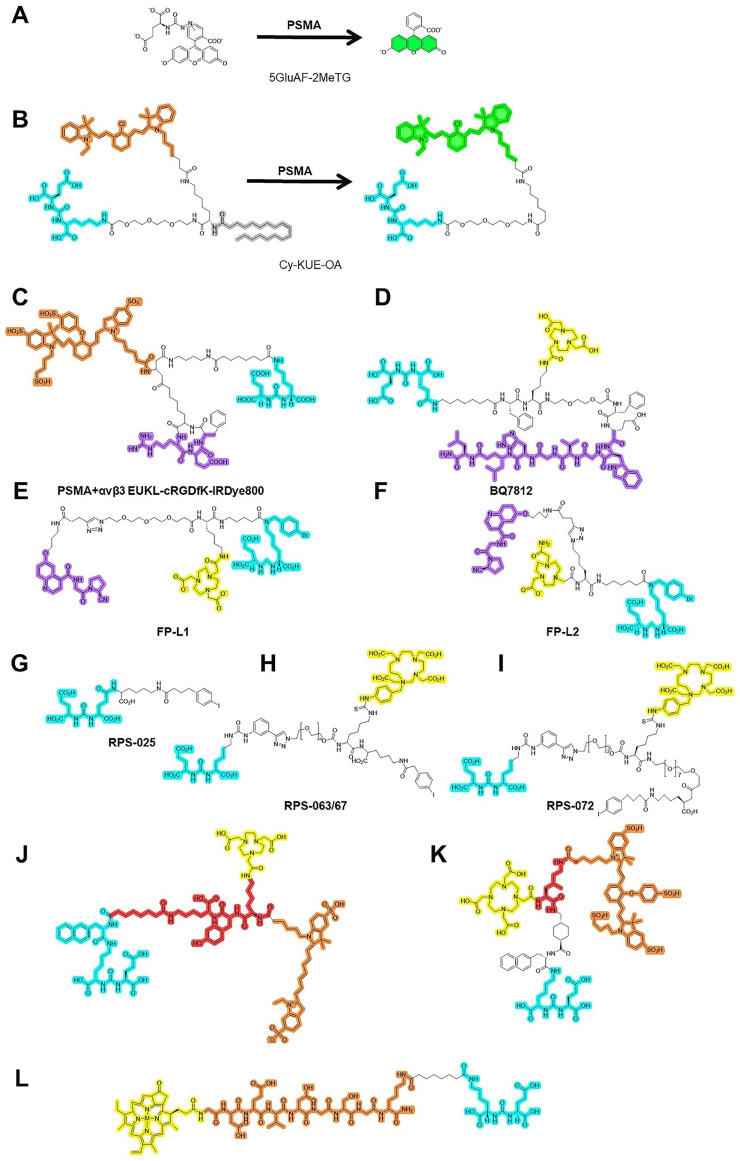
** Chemical structures of agents in PSMA-targeted activatable/multi-modality/multi-targeted NIRF dyes.** (A-B) activatable PSMA-targeted NIRF dyes; (C-F) Multi-modality PSMA-targeted dyes; (G-L) Multi-targeted PSMA-targeted dyes; PSMA ligands for PSMA-FGS. Blue, yellow, green, red, orange, purple separately highlight the PSMA binding motif, chelated area for the labeled radioisotope, activated NIRF area, linker, labeled near-infrared fluorescent probe, and binding motif for another molecule.

**Figure 4:**
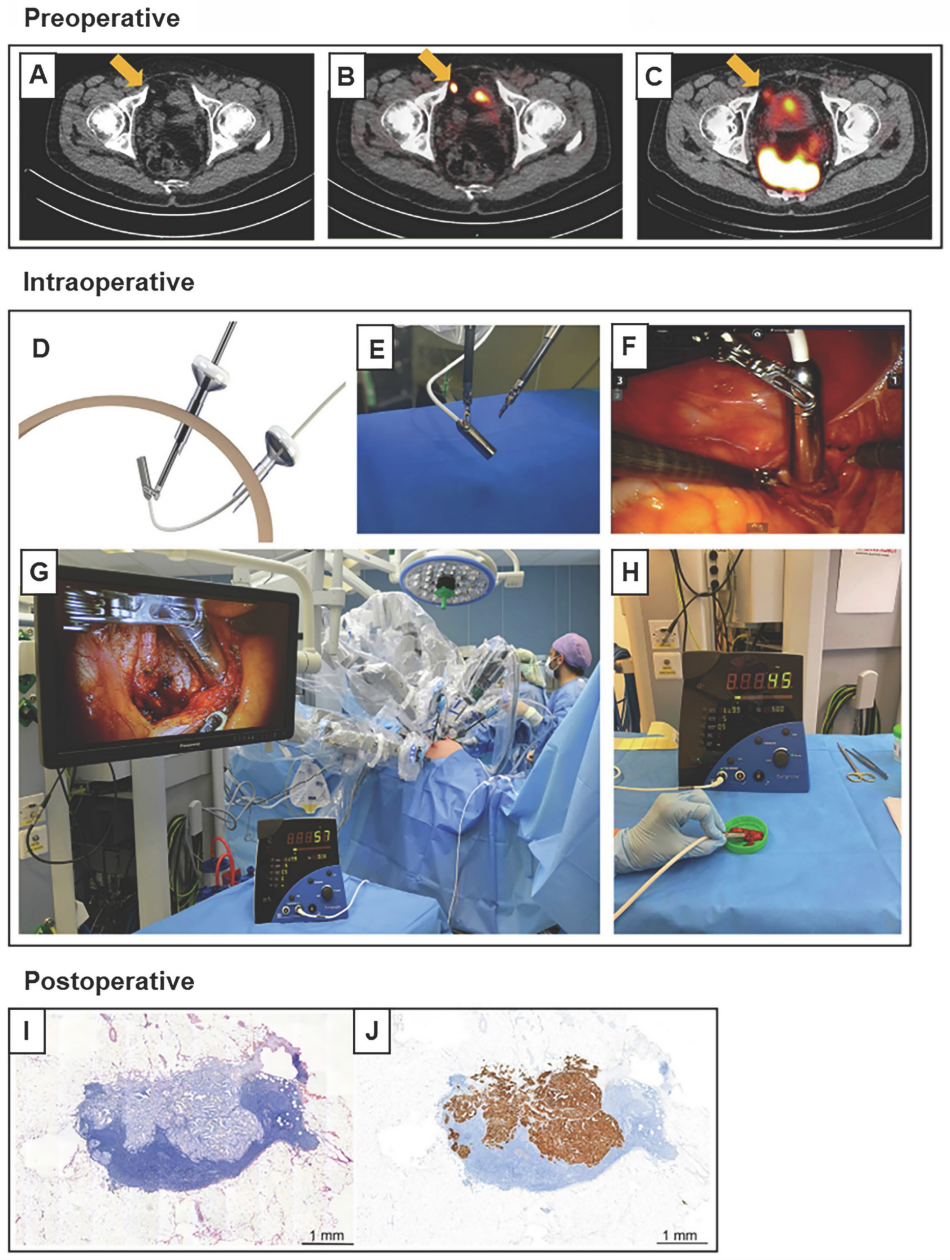
** Robot-assisted ^99m^Tc-PSMA radio-guided surgery procedure.** Preoperative (A) CT, (B) PSMA PET/CT, and (C) ^99m^Tc-PSMA-I&S SPECT/CT imaging demonstrate a prevesical lesion (indicated with an arrow) in a patient with biochemical recurrence after primary treatment for prostate cancer. (D) During the surgery, the DROP-IN probe is inserted in the abdomen through a trocar (E) and is autonomously maneuvered by the surgeon using a Da Vinci surgical console. (F, G*) In vivo* radioactivity measurement of a suspected prostate cancer recurrence. (H) *Ex vivo* radioactivity measurement of the resected specimen. Histological analysis revealed a lymph node metastasis on (I) hematoxylin and eosin staining and (J) PSMA immunohistochemistry. CT = computed tomography; PET = positron emission tomography; PSMA = prostate-specific membrane antigen; SPECT = single-photon emission CT. The figures were adapted with permission from 52, copyright 2022 European Urology © Elsevier, Inc.

**Figure 5:**
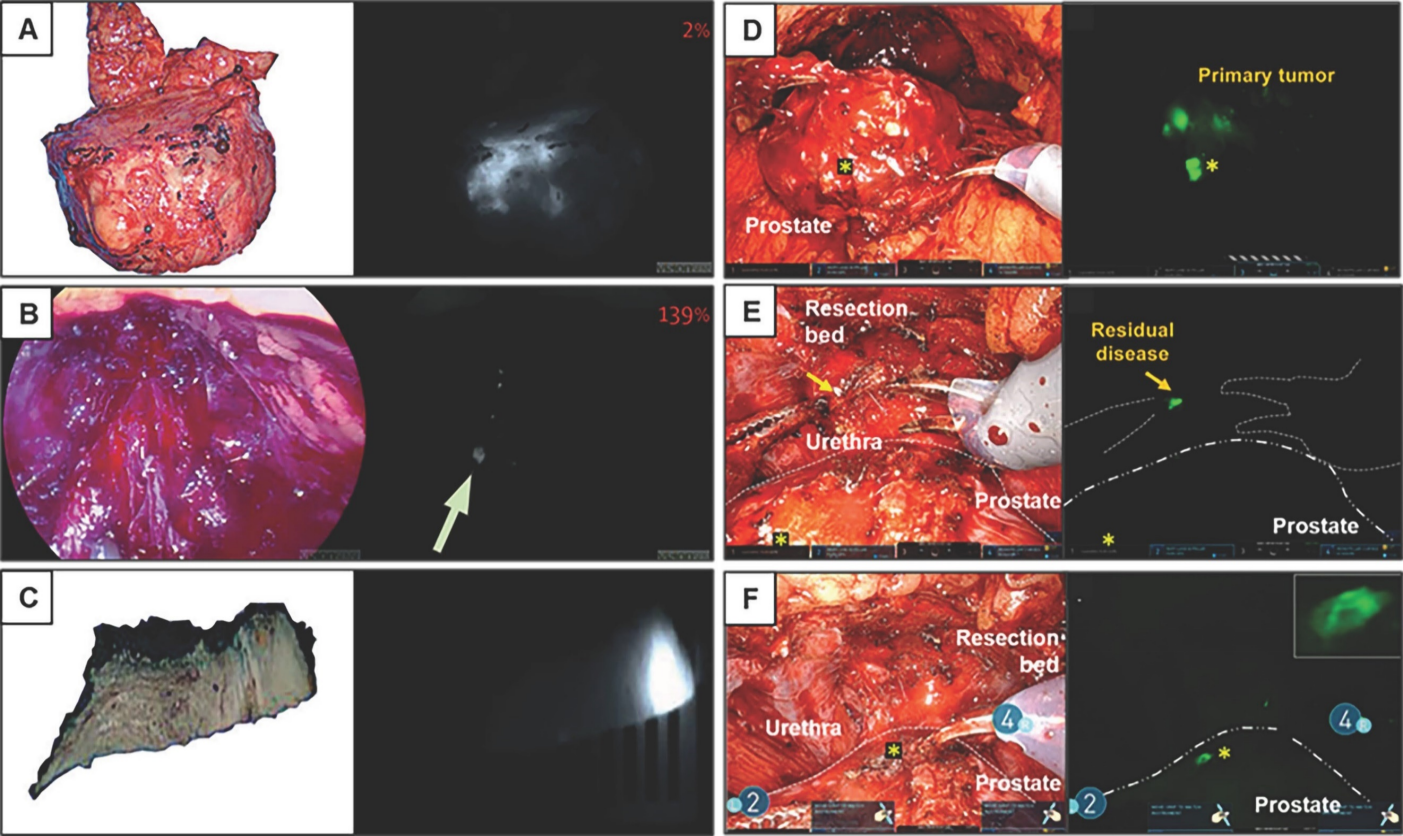
** Fluorescence workflow for surgical margin in apical prostate resection of OTL78(A-C) and IS-002 (D-F).** (A) The entire prostate is imaged directly after resection *ex vivo* on the backtable. Increased fluorescence signal is visible at the apex 15. (B) Subsequent assessment of the resection bed shows residual fluorescence signal (indicated with an arrow) where the apex used to be 15. (C) A fluorescent, PSMA-expressing positive surgical margin is seen on *ex vivo* gross macroscopy 15. (D) White light and sensitive Firefly image of the excised prostate 24 h after IS-002 injection (100 lg/kg) showing a strong, focal fluorescent signal in the primary tumor 16. (E) White light and sensitive Firefly images of an area where residual disease was identified in the anterior apical prostate resection bed near the urethra. Sensitive Firefly pinpointed (Fig. 2E) highly specific IS-002 fluorescent signal in the resection bed not deemed suspicious in white light endoscopy that was subsequently biopsied 16. (F) White light and sensitive Firefly images after biopsy. Insert in Figure 1F shows a strong fluorescent signal in the biopsied tissue, which was confirmed tumor positive following frozen sectioning. Further inspection of the prostate surface revealed (Fig. 2E) a fluorescent signal corresponding to a transected focal tumor extension. Gold arrows indicate biopsy location; gold stars mark transected tumor extension on prostate surface in Figures 2D and 2F. Dotted white lines (Fig. 2E) indicate instrument and prostate boundaries 16. The figures(A-C) were adapted with permission from 15, 52, copyright 2022 Lancet Oncology © Elsevier, Inc. The figures (D-F) were adapted with permission from 16, copyright 2023 Eur Urol Oncol © Elsevier, Inc.

**Figure 6:**
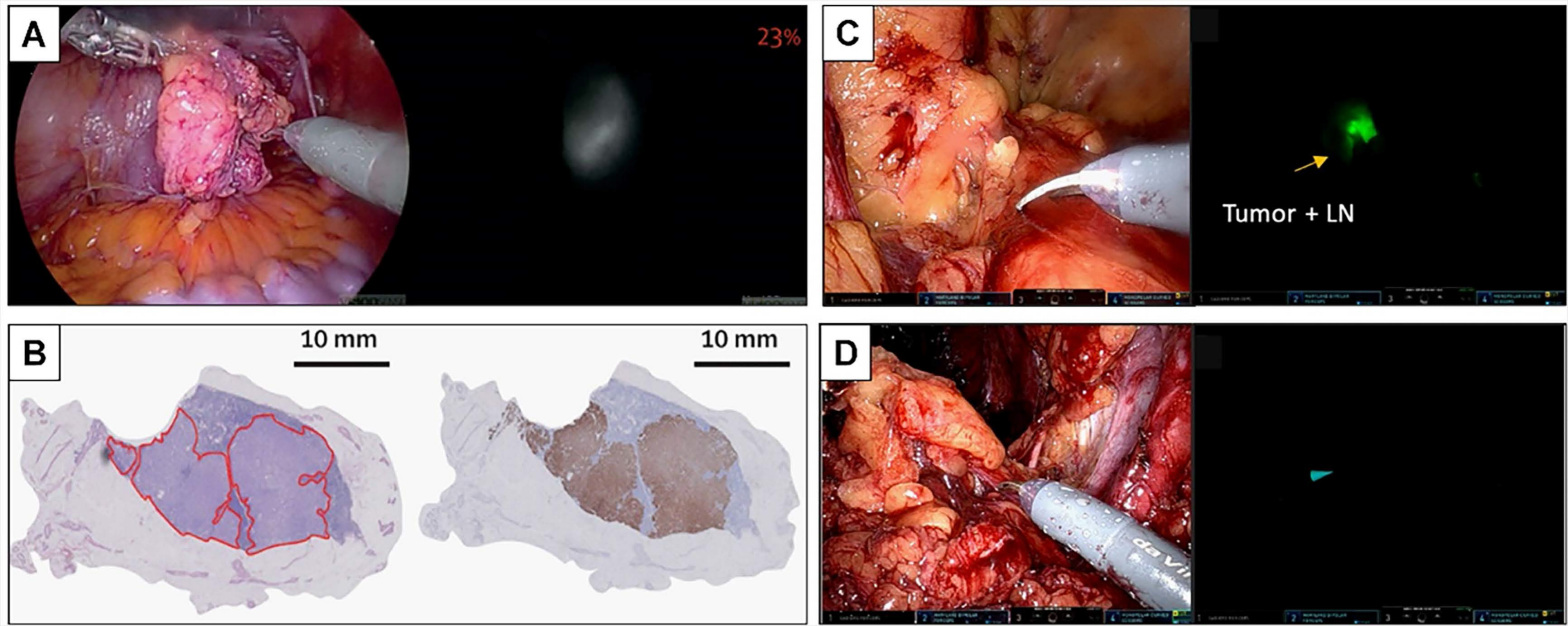
** Fluorescent imaging of a lymph node metastasis in PSMA-FGS of OTL78(A-B) and IS-002 (C-D).** (A) In-vivo bright-field, near-infrared and fluorescence color overlay imaging of a lymph node metastasis within a lymph node cluster using the VisionSense imaging system 15. (B) Corresponding haematoxylin and eosin tumor delineation, PSMA immunohistochemistry, and fluorescence imaging show a PSMA-positive metastatic lymph node with OTL78 uptake (OTL78 30 μg/kg). (C) White light endoscopy and sensitive Firefly image of tumor, positive lymph node for patient 2 (IS-002 25 μg/kg) 16. (D) Corresponding tumor-negative contralateral lymph node packages for patient 2 (IS-002 25 μg/kg) 16. The figures(A-B) were adapted with permission from 15, 52, copyright 2022 Lancet Oncology © Elsevier, Inc. The figures (C-D) were adapted with permission from 16, copyright 2023 Eur Urol Oncol © Elsevier, Inc.

PSMA also has glutamate carboxypeptidase (CP) activity. Activatable dyes, namely “turn on/turn off” agents, can highlight tumors with a lower background (Figure 3A-B). Kawatani *et al.* developed 5GluAF-2MeTG as the first in-class activatable NIRF dye for FGS patients with PCa 104 (Table 5). 5GluAF-2MeTG was designed based on previous findings that aryl glutamate conjugates with an azoformyl linker can be recognized by PSMA and have a sufficiently low lowest unoccupied molecular orbital energy level to quench the dye via photoinduced electron transfer 104. After incubation with recombinant human PSMA, 5GluAF-Fl showed significantly increased (>200 fold) fluorescence, which was mainly converted to highly fluorescent fluorescein 104. Xing *et al.* developed a self-quenching NIRF dye, Cy-KUE-OA, with dual PCa-membrane affinity. The targeted dye was anchored to the phospholipids of the cell membrane of PCa cells, dequenched, and showed strong Cy7 fluorescent light. In addition to *in vitro* and *in vivo* imaging in PSMA^+^ PCa tumor-bearing mice, the study further confirmed the performance of Cy-KUE-OA on surgically resected specimens of PCa tissues, LNMs, and healthy tissues from patients 105 (Table 5). Compared to previous preclinical studies, the validation of resected PCa specimens in this study was solid for clinical translation, providing a new method to validate the performance of dyes. The resected specimen validation in this study is an excellent method to validate the performance of dyes, bridging the gap between preclinical studies and clinical trials.

## 5. Multi-modality/multi-targeted PSMA-targeted surgery

With regard to the future development of PSMA-guided surgery, fluorescent and radioactive tracers are complementary and used in combination with multi-modality-guided surgery 106. Because PSMA-RGS and PSMA-FGS have their own strengths and shortcomings, multi-modality PSMA-targeted dyes with radiotracers and fluorescent dyes in a hybrid tracer were simultaneously developed for PSMA-RGS and PSMA-FGS (Figure 3 C-F). Integrating radioactive and fluorescent signatures into a single dye can enable surgeons to improve their intraoperative performance. The PSMA-11-based multi-modality agent ^68^Ga-Glu-urea-Lys-HBED-CC-IRDye800CW (^68^Ga-PSMA-11-IRDye800CW) also achieved accurate intraoperative detection of PSMA^+^ PCa tumors in tumor-bearing mice and healthy pigs 107. To overcome the nonspecific accumulation of PSMA-11 in non-malignant tissues, a histidine- (H) and glutamic acid (E)-containing linker ((HE)_3_-linker) was introduced between the chelator and the PSMA-binding motif 108. Compared to ^68^Ga-PSMA-11-IRDye800CW, ^68^Ga-Glu-urea-Lys-(HE)3-HBED-CC-IRDye800CW (^68^Ga-PSMA-914) can be used for preoperative PET imaging and PSMA-guided PARP with faster excretion and a lower background signal 109.

Another agent, NYM016, was designed using the NIRF dye Cy7 and NOTA chelator for ^68^Ga labeling. ^68^Ga-NYM016 was evaluated in a patient with recurrent PCa with metastatic lesions, showing an SUV_max_ of 18.93 in recurrent lesions 110. Furthermore, Aras *et al.* synthesized a multimodality agent, ^18^F-BF3-Cy3-ACUPA, and determined its safety and feasibility in 10 PCa patients by preoperative PET and intraoperative FGS 111. Harmatys *et al.* designed ^64^Cu-LC-Pyro, a low molecular weight PSMA-targeted agent with long plasma circulation time and high tumor-to-background ratio (10:1) for PET/CT, NIRF imaging and NIR-PIT at the same time 112.

Cordonnier *et al.* designed an oleic acid-coated spherical NaYF4:Yb,Tm@NaYF4 core/shell upconversion nanoparticle (UCNP) labeled with ^125^I for PSMA-targeted FGS/RGS in LNCaP-Luc-bearing mice 113. Cheng *et al.* developed a theranostic nanotexaphyrin for PSMA targeted SPECT/CT imaging and PIT through the chelation of metal isotopes (^111^In/^175^Lu), achieving a TBR of 2.7 114. Although the multi-modality method combines the strengths of PSMA-RGS and PSMA-FGS, challenges have also been exemplified. For instance, the diagnostic standard for PCa lesions and LNMs in multi-modality PSMA-targeted surgery, decisions regarding contradictory results of PSMA-RGS and PSMA-FGS, and the hybrid modality weakening the performance of each imaging modality remain to be elucidated. Radiation and contradictory results from multi-modality imaging may limit its further development and clinical translation.

It is well known that PSMA expression is heterogeneous and can vary between patients, lesions in the same patient, or even within the same lesion. To better recognize all LNMs during PSMA RGS, multi-targeting methods have also been attempted (Figure 3G-K). Integrin αvβ3 is a reasonable co-target with PSMA for neo-vasculature which incorporates the Arg-Gly-Asp (RGD) binding motif. EUKL-cRGDfK-IRDye800 could be used to illuminate either the PSMA^+^ or integrin αvβ3^+^ xenografts in mice 115. Dual-targeting agents with PSMA-targeting ability and complementary molecules have also been introduced 116. PSMA and gastrin-releasing peptide receptor (GRPR) are complementarily expressed in PCa tumors, as has been validated in prostatectomy samples and patients 117-119. For targeting GRPR, RM-26 (GRPR antagonist) and its dual-targeting radiotracers (RGD-RM26-03, denoted as LNC1015) have showed favorable pharmacokinetics and high clinical feasibility for PET/CT imaging of cancer 120-124. Similarly, Schollhammer *et al.* also retrospectively studied 20 frozen samples with various GS group using ^111^In-PSMA-617 and GRPR antagonist ^111^In-RM2^117^. They found that ^111^In-PSMA-617 had a higher uptake in the higher GS group, whereas ^111^In-RM2 had higher binding in the lower GS group 117. The dual-tracer method was also validated in recent clinical studies. Qiu *et al.* also showed that dual-tracer (NOTA-P2-RM26 and PSMA-617) PET/CT-targeted biopsy (TB) achieved a higher PCa detection rate (69.77%) than systematic biopsy SB (29.29%) or mpMRI-TB (36.14%) 125. GRPR/PSMA TB is better than GRPR TB or PSMA TB alone: the csPCa omission diagnostic rate of dual-tracer PET/CT-TB (15.38%) was significantly lower than ^68^Ga-GRPR PET/CT-TB (48.72%) or ^68^Ga-PSMA PET/CT-TB (28.21%) 125. Recently, a PSMA/GRPR-targeting radioligand named ^68^Ga-BQ7812 was also developed but just in preclinical stage 126. This compound holds promise for increasing the chance of specific tumor binding, thereby improving detection accuracy, especially in patients with heterogeneous expression of either target. Fibroblast activation protein alpha (FAP) is a transmembrane protease highly expressed in the tumor microenvironment of PCa 127. Boinapally *et al.* developed small-molecule PSMA- and FAP-targeting moieties with ^64^Cu, named ^64^Cu-FP-L1 and ^64^Cu-FP-L2, respectively 116, with the potential to be used for PSMA-FAP dual-targeted guided PCa surgery. These PSMA/FAP dual-targeted radioligands enable imaging of lesions expressing FAP, PSMA, or both on the tumor cell surface or within the tumor microenvironment. These multi-targeted dyes were developed for patients with varying levels of PSMA expression in PCa tumors, including those with high, low, or no PSMA expression. While as nearly all treatment-naïve patients receiving PSMA-RGS had PSMA-positive PCa tumors expressing 10-100-fold more PSMA than surrounding benign prostatic tissues 5 and loss of PSMA expression is commonly observed in patients with CRPC who do not usually undergo further surgery, a well-performing PSMA single-targeted dye may suffice for most clinical needs in PSMA-RGS or PSMA-FGS, multi-targeted conjugates offer promise for patients with PCa tumors displaying low or reduced PSMA expression. These conjugates provide additional advantages for managing heterogeneous tumors.

**Figure 7:**
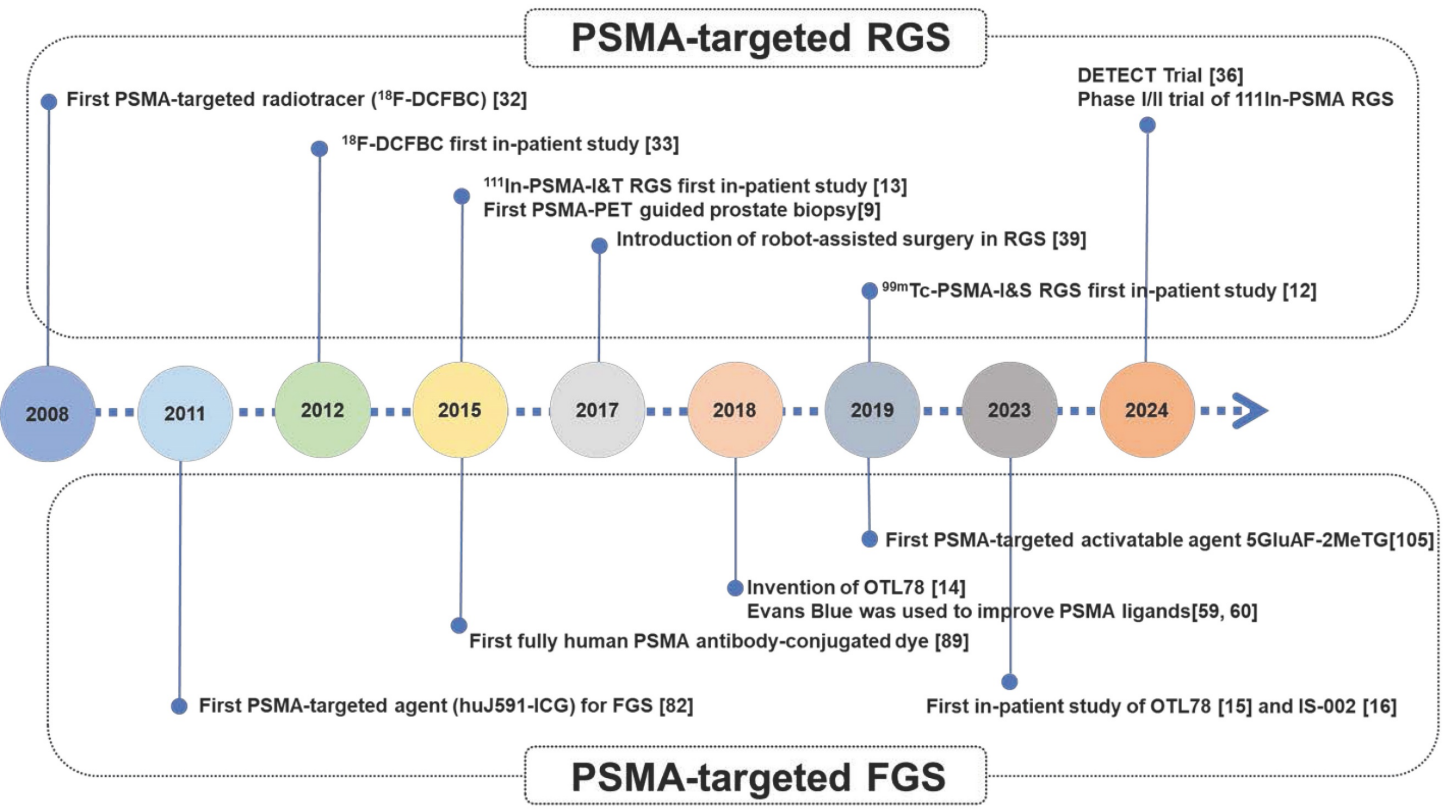
Chronological timeline highlighting major events and significant agents for PSMA-targeted guided surgery.

Thus, we believe that establishment and clinical validation of a well-performing PSMA-targeted dye (such as LNC1003) with reliable safety and a long operation time window can meet the greatest clinical need for PSMA-targeted surgery. For a better understanding of the development of PSMA-targeted surgery, a chronological timeline highlighting major events and significant agents for PSMA-targeted guided surgery was shown in Figure 7.

## 6. Conclusions

PSMA-targeted surgery can lead to significant changes in traditional surgical procedures for PCa because of its high sensitivity in the detection of PSMs and micro-LNMs compared to any preoperative imaging or visualization. PSMA-targeted surgery may benefit PCa patients with PSMA-TB because of a more accurate pathological diagnosis by PSMA-RGS or PSMA-FGS for a more accurate resection of all prostatic and metastatic lesions. PSMA-TB, PSMA-RGS, and PSMA-FGS should be used as overall strategies for patients with primary csPCa or recurrent PCa, thereby greatly facilitating clinical diagnosis and surgical treatment. Among the above strategies, PSMA-RGS has entered clinical use in many trials, but we believe that PSMA-FGS will be a developing trend in the near future, as it is radiation-free and intuitively used in real-time within surgery. The multi-targeted method may be more suitable for CRPC radiotherapy than PSMA-targeted surgery.

Multi-modality methods have strengths, but the shortcomings of both agents and contradictory results may limit their use. Importantly, the close correlation among preoperative imaging, PSMA-TB, PSMA-FGS, and follow-up evaluations after surgery requires further investigation. However, a well-performing clinical standard to select the most suitable candidates for PSMA-RGS and PSMA-FGS is still lacking. More attention should be paid to potential PSMA-FGS dyes, and their efficacy should be validated in more clinical trials in the near future. With the rapid development of PSMA-targeted dyes, PSMA-targeted surgery, and its related standard flow would completely change conventional surgical methods and improve the level of precision surgery for all patients with PCa.

## Figures and Tables

**Table 1:** Inclusion Criteria of patients for PSMA-RGS.

Year	Country	Inclusion Criteria
**Primary PCa**		
2015^13^	Germany	Primary PCa and suspicion of recurrent LNMs on preoperative PSMA PET/CT
2018[38], 2021[49]	Germany	Suspicion of LNMs on preoperative PSMA PET/CT
2022^50^	Italy	Intermediate- or high-risk cN0cM0 PCa at conventional imaging with a risk of LNI of >5% (Briganti nomogram risk)
2022^51^	Australia	Primary high-risk PCa (≥cT3a, international society of urological pathology (ISUP) Grade Group ≥3 or PSA of ≥ 15 ng/mL) with potential LNMs (Briganti nomogram risk >10% or on preoperative imaging)
**Recurrent PCa**		
2016^37^	Germany	Rising PSA level to ≥0.2 ng/mL after primary curative therapy;^68^Ga-PSMA-11 PET-positive soft tissue lesions
2018^45^	Germany	Recurrent PCa on preoperative PSMA PET/CT
2022^42^	Netherlands	Up to three pelvic PCa recurrences (nodal or local) on preoperative PSMA PET/CT
2022^54^	Germany	Recurrent PCa after initial RP with positive LNMs on preoperative PSMA PET/CT
2023^42^	Germany	Patients with positive lesion detected on PSMA PET imaging in the pelvis or retroperitoneum suspicious for lymph node metastases (LNMs) or local recurrence.
2023^69^	Germany	Conforming initial treatment with either radical prostatectomy (RPE) or curatively intended external beam radiation, prostate-specific antigen (PSA) ≥0.5 ng/mL, positive ^68^Ga-PSMA I&T PET/CT scan with no more than 2 intense PSMA-positive lymph nodes (maximum uptake at least 6 kBq/mL), and no signs of local recurrence or distant metastases, as well as informed consent to participate in this study.

**Table 2:** Isotopes and relevant characteristics for PSMA-targeted radionuclides.

Isotopes	Agents	Imaging/therapeutic properties	Half-lives	Stage	Strengths	Limitations
Commonly used						
^111^In	^111^In-PSMA-I&T; ^111^In-PSMA-617	Imaging-PSMA-RGS	2.83 d	Clinical trial	Earliest used agents	Suboptimal nuclear properties, high cost, and limited availability of ^111^InCl_3_
^99m^Tc	^111^In-PSMA-I&S	Imaging-PSMA-RGS	6.02 h	Clinical trial	Optimal nuclear properties, lower cost, and easily available	Inferior ability in pre-operative imaging, compared with PET/CT
^177^Lu	^177^Lu-PSMA-617; ^177^Lu-PSMA-I&T	Beta-therapy	6.65 d	Clinical trial	Most widely used agents for therapy of CRPC	Heterogeneous PSMA expression after ADT
^225^Ac	^225^Ac-PSMA-617	Alpha-therapy	10.00 d	Clinical trial	Higher level of radiobiologic effectiveness when compared with Beta-therapy	Heterogeneous PSMA expression after ADT
^18^F	^18^F-DCFPyL	PET/CT	110 min	FDA approved 2021	High resolution	Relatively limited availability
^68^Ga	^68^Ga-PSMA-I&T; ^68^Ga-PSMA-11	PET/CT	6.7 h	FDA approved 2020 (PSMA-11)	High resolution	Relatively limited availability
Uncommonly used						
^44^Sc	^44^Sc-PSMA-617	PET/CT	3.93 h	Clinical trial	More appropriate for delayed imaging	——
^64^Cu	^64^Cu-CA003;^ 64^Cu-CA003	PET/CT	12.7 h	Preclinical	——	Instability of ^64^Cu complexes in vivo
^89^Zr	^89^Zr-PSMA-DFO	PET/CT	78.4 h	Clinical trial	Localize lesions with weak PSMA expression	——
^131^I	^131^I-MIP-1095	Beta-therapy	8.02 d	Clinical trial	A potential alternative to ^177^Lu-PSMA	——
^161^Tb	^161^Tb-PSMA-617	Alpha-therapy	7.90 d	Preclinical	A potential alternative to ^177^Lu-PSMA	——
^165^Er	^165^Er-PSMA-617	Alpha-therapy	10.40 h	——	——	——
^212^Pb	^212^Pb-CA012	Alpha-therapy	10.64 h	Preclinical	A potential alternative to ^225^Ac-PSMA	——
^213^Bi	^213^Bi-PSMA-617; ^213^Bi-PSMA I&T; ^213^Bi-JVZ-008	Alpha-therapy	45.59 min	Clinical trial	A potential alternative to ^225^Ac-PSMA	——
^227^Th	^227^Th-PSMA-TTC (BAY 2315497)	Alpha-therapy	18.70 d	Preclinical	Synergistic antitumor efficacy in combination with darolutamide	——

**Table 3:** PSMA-RGS in PCa patients.

Year	Country	Retro/pros	Patient number	Patient type	Preoperative staging	Gamma probe	Agents for PSMA-RGS	Age	tPSA (ng/ml)	GS	sensitivity	specificity	PPV	NPV	Accuracy
2015^13^	Germany	R	5	Primary PCa; Recurrent PCa	68Ga-PSMA-11 PET/CT	Crystal Probe CXS-SG603	111In-PSMA-I&T	75 (IQR 64-75)	2.45 (IQR 0.46-4.36).	——	100.0%	100.0%	100.0%	100.0%	100.0%
2016^37^	Germany	R	31	Recurrent Pca	68Ga-PSMA-11 PET/CT	Crystal Probe CXS-SG603	111In-PSMA-I&T	68.2 (Range 52-76)	1.3 (IQR 0.57-2.53)	7 (≤6-10)	92.3%	93.5%	88.9%	95.6%	93.1%
2018^38^	Germany	R	6	Primary Pca	68Ga-PSMA-11 PET/CT	Ex situ gamma probe	111In-PSMA-617	71 (IQR 66.5-75.5)	7.03 (IQR 1.31-12.02)	7 (7-9)	92.1%	98.9%	94.6%	98.4%	97.7%
2018^45^	Germany	R	31	Recurrent PCa	68Ga-PSMA-11 PET/CT	Crystal Probe CXS-SG603	99mTc-PSMA-I&S	66.7(IQR 60.5-73.5)	1.13 (IQR: 0.71-2.35)	7 (5-9)	83.6%	100.0%	100.0%	89.2%	93.0%
2021^49^	Germany	R	6	Primary PCa; Recurrent PCa	68Ga-PSMA-11 PET/CT; 18F-PSMA-1007	In situ/Ex situ gamma probe	99mTc-PSMA-I&S	64(IQR 54.7-67.3)	33.3 (IQR: 4.6-64.4)	8 (7b-9)	76.6%	94.4%	89.4%	86.9%	——
2022^42^	Netherlands	P	20	Recurrent Pca	68Ga-PSMA-11; 18F-DCFPyl PET/CT	DROP-IN Gamma Probe	99mTc-PSMA-I&S	68 (IQR 66-72)	1.02 (IQR 0.46-2.43).	——	86.0%	100.0%	100.0%	95.0%	80.0%
2022^50^	Italy	P	12	Primary PCa	68Ga-PSMA-11 PET/MRI	DROP-IN Gamma Probe	99mTc-PSMA-I&S	70 (IQR 66-71)	8.7 (IQR: 4.8-15.5)	8 (7a-9)	63.0%	99.0%	83.0%	96.0%	——
2022^51^	Australia	P	12	Primary PCa	68Ga-PSMA-11 PET/CT	DROP-IN gamma probe	99mTc-PSMA-I&S	68 (IQR 57-69)	9.15 (IQR: 6.0 - 21.2)	9 (8-9)	76.0%	69.0%	50.0%	88%,	——
2022^54^	Germany	R	364	Recurrent Pca	PSMA PET/CT	Crystal Probe CXS-SG603	111In-PSMA-I&T or 99mTc-PSMA-I&S	67 (IQR 62-71)	1.0 (IQR: 0.5 - 1.9)	7a (6-≥9)	——	——	——	——	——
2023^42^	Germany	P	85	Recurrent Pca	PSMA PET/CT	Crystal Probe CXS-SG603	99mTc-PSMA-I&S	63 (IQR 60-69) Open; 64 (IQR60-67) Robot	0.58 (0.38, 0.87) Open; 0.44 (0.31, 0.71) Robot	——	——	——	——	——	——
2023^69^	Germany	P	6	Recurrent Pca	68Ga-PSMA I&T PET/CT	Crystal Probe CXS-SG603	67Ga-PSMA I&T	69.5 (IQR 63-73)	2.7 (IQR: 1.125 - 3)	7a (6-9)	——	——	——	——%	——
2024^36^	Netherlands	p	20	Primary PCa	18F-PSMA PET/CT	DROP-IN gamma probe	111In-PSMA-I&T	69 (Range 57-79)	22.2 (2.9-117)	——	66.7%	99.8%	96.8%	97.0%	——

**Table 4:** PSMA-FGS in PCa patients.

Year	Stage	Country	Institution	Excitation/emission	NCT#	Agents	Patient number	Patient Type	FGS type
2023^15^	Phase 2a	Netherlands	Leiden University Medical Center, Leiden University, Leiden	774/— nm	European Trial Database, 2019-002393-31;	OTL78	18	Primary PCa with ISUP≥7	RP±ePLND (da Vinci surgical system)
2023^16^	Phase I	USA	University of California, San Francisco	774/793 nm	NCT04574401	IS-002	24	High-risk primary prostate cancer	RP+ePLND (da Vinci surgical system)

**Table 5:** Potential agents for PSMA-FGS in the preclinical stage

Year	Country	Institution	Type of ligands	Agents	Aim	In-vivo experiment
2011^82^	USA	National Cancer Institute	Humanized Antibody	ICG-huJ591	FGS	PC3±PSMA
2016^83^	USA	Johns Hopkins University School of Medicine	Humanized Antibody	J591-IR800	FGS	PC3±PSMA; LMD±PSMA
2017^84^	USA	National Cancer Institute	Fully human antibody	Anti-PSMA-IR700	FGS/PIT	PC3±PSMA
2019^87^	China	Fourth Military Medical University	Fully human antibody	PSMAb-IRDye800CW	FGS	PC3±PSMA
2016^88^	China	Fourth Military Medical University	scFv	gy1-IRDye800CW	FGS	PC3±PSMA
2016^89^	Italy	Verona University	scFv	X770-scFvD2B	FGS	LNCaP
2019^90^	Netherlands	Radboud university medical center	scFv	111In-DTPA-D2B-IRDye700DX	FGS/PIT	LS174T±PSMA
2010^91^	USA	Washington State University	PSMA inhibitor CTT-54.2	Cy5.5-CTT-54.2	FGS	LNCaP; PC3
2009^92^	USA	Johns Hopkins Medical Institutions	PSMA inhibitor YC-27 3	YC-27 3-IRDye800CW	FGS	PC3±PSMA
2014^93^	USA	Case Western Reserve University	PSMA inhibitor PSMA-1	PSMA-1-IRDye800CW; PSMA-1-Cy5.5	FGS	PC3±PSMA
2017^85^	USA	Johns Hopkins Medical Institutions	IRDye700DX	YC-9	FGS	PC3±PSMA
2020^86^	Canada	University Health Network	PSMA inhibitor	BPP	FGS	PC3±PSMA
2019^14^	USA	On Target Laboratories	PSMA inhibitor	OTL78	FGS	LNCaP;22Rv1; PC3; A549
2017^94^	Japan	Yoshida Shimoadachi-cho	PSMA inhibitor	800CW-SCE	FGS	LNCaP; PC3
2017^95^	USA	Massachusetts General Hospital and Harvard Medical School	PSMA inhibitor KUE	ZW800-1	FGS	LNCaP; PC3
2018^112^	Canada	University Health Network	PSMA inhibitor	64Cu-LC-Pyro	PET/CT FGS	PC3±PSMA
2022^96^	USA	Johns Hopkins Medical Institutions	PSMA inhibitor	DyLight800-10	FGS	PC3±PSMA
2022^99^	Germany	German Cancer Research Center (DKFZ)	PSMA inhibitor PSMA-617	PSMA-927	FGS	LNCaP; PC3
2022^102^	Netherlands	Radboud University Medical Center	PSMA inhibitor PSMA-N064	PSMA-N064inc	FGS/PIT	LS174T±PSMA
2022^103^	Netherlands	Leiden University Medical Center	PSMA inhibitor EuK	99mTc-EuK-(SO3)Cy5-mas3	FGS	Porcine model (15 non-tumor-bearing pigs)
2023^105^	China	Peking Union Medical College and Chinese Academy of Medical Sciences	PSMA inhibitor KUE	Cy7	FGS	C4-2;22RV1; PC3
2022^100^	UK	King's College London	PSMA inhibitor urea-based Glu-Lys	Cy7.5	FGS	LNCaP; PC3; DU145; DU145
2023^97^	China	Northwestern Polytechnical University	PSMA inhibitor Lys-urea-Glu	iridium(III) complex	FGS	LNCaP; 22Rv1; PC3
2022^101^	Italy	University of Torino	PSMA-617	IRDye700DX	FGS	LNCaP; PC3
2022^104^	Japan	The University of Tokyo	Activatable	5GluAF-2MeTG	FGS	LNCaP; PC3

## Abbreviations

RP: Radical prostatectomy
PLND: pelvic lymph node dissection
PCa: prostate cancer
PSMA PET: Prostate specific membrane antigen positron emission tomography
PSMA: prostate-specific membrane antigen
PSMs: positive surgical margins
LNMs: lymph node metastases
PSMA-TB: PSMA PET guided prostatic biopsy
PSMA-RGS: PSMA targeted radio-guided surgery
PSMA-FGS: PSMA targeted fluorescence-guided surgery
FOLH1: Folate hydrolase 1
GCP II: Glutamate carboxypeptidase II
NP: normal prostate
111In-PSMA-I&T: 111Indium-PSMA imaging and therapy
99mTc-PSMA- I&S: 99mTechnetium-PSMA imaging and surgery
csPCa: clinically significant prostate cancer
mpMRI: multiparametric magnetic resonance imaging
PB: prostate biopsy
RARP: robot-assisted radical prostatectomy
PSA: prostate-specific antigen
PPV: positive predictive value
NPV: negative predictive value
OT: operating time
EBL: equal estimated blood loss
cBR: Complete biochemical response
MAS3: mercaptoacetyl triserine
ePLND: extended pelvic nodal dissection
TFS: therapy-free
SPECT/CT: Single-Photo Emission Computed Tomography/Computer Tomography
CP: carboxypeptidase
GRPR: gastrin-releasing peptide receptor
